# Burden and impact of periodontal diseases on oral health-related quality of life and systemic diseases and conditions: Latin America and the Caribbean Consensus 2024

**DOI:** 10.1590/1807-3107bor-2024.vol38.0117

**Published:** 2024-11-22

**Authors:** Giuseppe Alexandre ROMITO, James Rudolph COLLINS, Mohamed Ahmed HASSAN, Carlos BENÍTEZ, Adolfo CONTRERAS

**Affiliations:** (a) Universidade de São Paulo – USP, School of Dentistry, Department of Periodontics, São Paulo, SP, Brazil.; (b) Pontificia Universidad Católica Madre y Maestra, School of Dentistry, Department of Periodontology, Santo Domingo, República Dominicana.; (c) Colgate-Palmolive, Piscataway, NJ, USA.; (d) Universidad del Valle, School of Dentistry, Discipline of Periodontics, Cali, Colombia.

**Keywords:** Periodontal Diseases, Quality of Life, Chronic Disease, Oral Health, Latin America, Caribbean Region

## Abstract

Periodontal diseases are highly prevalent globally, and represent a significant public health burden that could affect the quality of life in Latin American and in Caribbean countries and territories. The primary objective is to explore the existing research and epidemiological studies on the burden of periodontal diseases, particularly their impact on oral health-related quality of life (OHRQoL) and associations with systemic health conditions in Latin America and the Caribbean (LAC). An electronic literature search was conducted across multiple databases, including MEDLINE (PubMed), Scopus, LILACS, SciELO, and Web of Science, without publication date or language limitations, up until December 2023. Reviewers independently assessed titles and abstracts based on the eligibility criteria. The search yielded 1195 articles, with 63 meeting the inclusion criteria. The results of epidemiological studies showed that periodontitis is extremely prevalent at 90% in LAC; severe periodontitis can affect nearly 10% of the adult population and that periodontitis is aggravated by smoking, poverty, low education level, and limited access to proper dental care. Periodontitis was consistently associated with worse OHRQoL; and causing pain, and/or triggering psychological discomfort, physical disability, and social disability. Associations were also reported between periodontitis and comorbidities such as diabetes, cardiovascular disease, rheumatoid arthritis, respiratory disease, mental illness, and adverse pregnancy outcomes that are also affecting the quality of life of individuals and their families. This scoping review offers a thorough examination of the burden of periodontal diseases in LAC and highlights the significant public health concern that it represents for the region.

## Introduction

Gingivitis and periodontitis are highly prevalent conditions worldwide and represent a major public health burden. They are the most common chronic inflammatory non-communicable disease (NCD) in humans.^1^ In Latin American adolescents from 15 to 18 years of age, the prevalence of CAL ≥ 3 mm is 32.6%, and the prevalence of the probing pocket depth ≥ 4 mm is 59.3%.^2^In addition, the prevalence of severe periodontitis in adults reached 7.8% to 25.9% from middle aged adults to seniors, respectively.

Epidemiological studies have shown that the prevalence and severity of periodontal diseases are high, commonly exceeding 90% of the population, if gingivitis is included.^2^ Periodontitis has been associated with poor oral health-related quality of life (OHRQoL), with detriments found in multiple domains, including pain, psychological discomfort, physical disability and social disability.^3^ There is also evidence of associations between periodontitis and systemic health conditions that are also prevalent in the region, including diabetes, cardiovascular disease,^4^ rheumatoid arthritis,^5^ respiratory disease,^6^ mental illness,^7,8^ and adverse pregnancy outcomes.^9^

The high burden of disease, combined with the impact on quality of life and associations with chronic diseases, emphasizes the need to prioritize periodontal health as an essential public health issue since it is affecting at least half of the 670 million inhabitants in Latin America and the Caribbean countries (LAC).^10^ Strategies to reduce this burden include improving oral hygiene practices throughout the life cycle of individuals and promote general health education campaigns, increasing access to professional dental care, incorporating screening and management of periodontitis into managing systemic diseases, and addressing common risk factors such as smoking, malnutrition and the pathophysiological complication of comorbidities.^2^

The rationale behind this consensus and scoping review stems from the recognition of periodontal diseases as a significant public health challenge in Latin America and the Caribbean (LAC). The widespread prevalence and severity of these conditions, coupled with their impact on Oral Health-Related Quality of Life (OHRQoL) and their association with systemic diseases, point out the need for a comprehensive review of existing evidence. Our objective is to elucidate the scope of the issue, evaluate current knowledge, identify gaps, and provide recommendations for future research and public health initiatives. This consensus underscores the urgent need for heightened attention to periodontal diseases in LAC and furnishes evidence to inform health promotion, disease prevention, and control strategies for periodontal disease and its most prevalent comorbidities.

## Methods

A scoping review was conducted in accordance with the PRISMA extension for Scoping Reviews (PRISMA-ScR). However, as this is an investigation to support the clinical consensus of periodontology in the region, this manuscript was not registered.

### Objectives of the scoping review

#### Primary objective

a.To explore existing research on the burden of periodontal diseases in Latin America and the Caribbean, with focus on their impact on oral health-related quality of life (OHRQoL) and associations with some systemic health conditions.

#### Secondary objectives

- To map the extent, range, and nature of the literature on periodontal diseases in Latin America and the Caribbean.
- Assess the impact of periodontal diseases on OHRQoL in these regions.
- Identify gaps in the current literature to inform future research directions.
- Provide evidence-based recommendations for public health strategies and interdisciplinary approaches to mitigate the impacts of periodontal diseases.

## Focused questions for the scoping review

Q1: What evidence is there with regard to the impact of periodontal diseases in Latin America and the Caribbean, specifically addressing their influence on oral health-related quality of life?

Q2: What evidence is available about the relationship between oral health-related quality of life and the association of these diseases with systemic diseases and conditions relative to the impact of periodontal diseases in Latin America and the Caribbean?

## Eligibility criteria

### The inclusion criteria

- Interventional and observational studies (e.g., randomized clinical trials, non-randomized clinical trials, cohort studies, cross-sectional studies)
- Studies that were conducted in Latin America and the Caribbean.
- Studies focused on periodontal diseases.
- Studies examining the impact on oral health-related quality of life.
- Studies exploring associations with systemic diseases and conditions.

### The exclusion criteria:

- Studies outside the geographical scope.
- Studies that were not related to periodontal diseases or quality of life.
- Studies with different design and scope (In-vitro, animal, literature reviews)

### Search strategy

**Figure 1:**
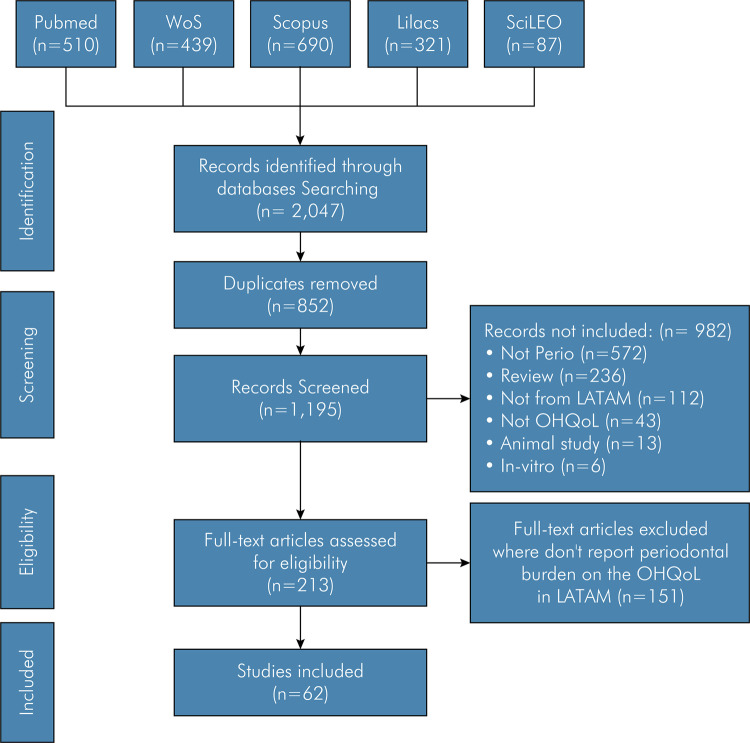
Study flow chart of oral health quality of life and periodontitis articles in Latin America and the Caribbean countries.

An electronic literature search was conducted across multiple databases, including MEDLINE (PubMed), Scopus, LILACS, SciELO, and Web of Science. Search strategies for each database were developed based on key terms to retrieve articles focused on the research questions. The main aim was to assess the burden of periodontal disease in combination with systemic diseases, conditions and its impact on the oral health-related quality of life (OHRQoL) of Latin American and Caribbean populations. Different research strategies were created, focused on every specific disease and condition that was reported in Figure 1. Languages of publication include English, Portuguese and Spanish and the date of publication considered was until December 2023.

## Study selection

Based on the eligibility criteria, two reviewers (MAH and CB) independently assessed the titles and abstracts of the studies identified by means of the search strategy. Any discrepancies in their assessments was resolved through discussion and, if necessary, by the intervention of a third reviewer (JC, GAR, or AC). Furthermore, (MAH, CB, and JC) evaluated the full texts of studies that met the inclusion criteria or those with ambiguous information in the title and abstract.

## Results

**Figure 2:**
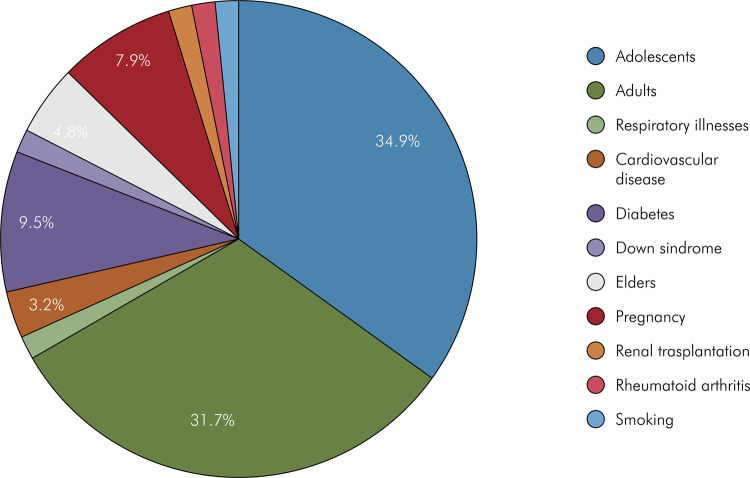
Proportion of articles related to periodontal disease, age of patient group, comorbidities and Quality of Life.

**Figure 3:**
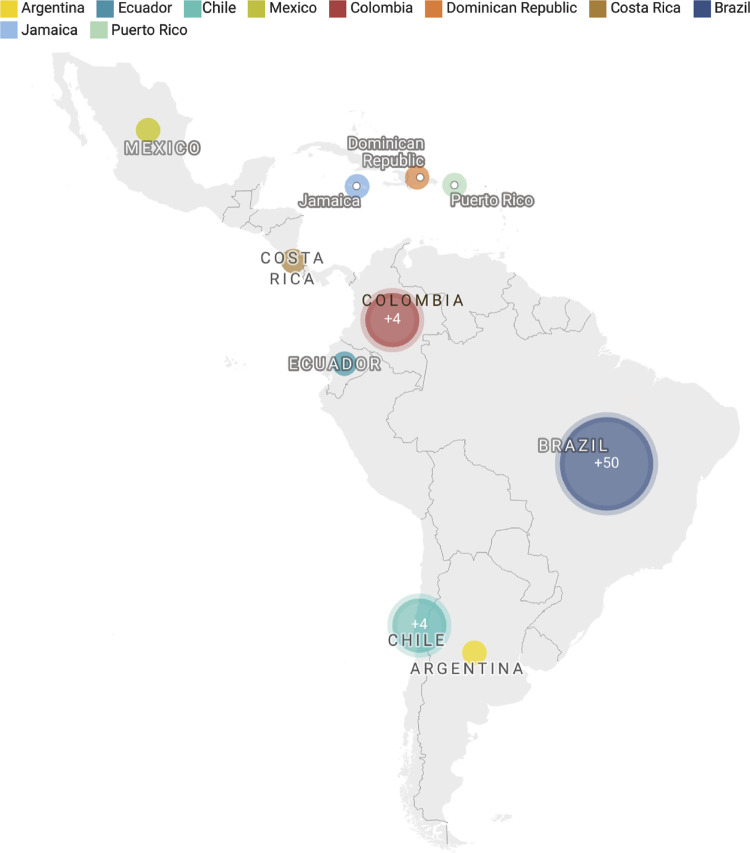
Distribution of articles by country, Brazil (n = 50), Colombia (n = 4), Chile (n = 4), Ecuador (n = 1), Dominican Republic, Jamaica, Puerto Rico (n = 1), Costa Rica (n = 1), Mexico (n = 1), Argentina (n = 1).

The electronic search yielded 1,195 articles. Of these, 63 met the inclusion criteria and were included (Figure 1). Among the articles included, 53 were in English, 8 in Portuguese, and 2 in Spanish. These articles reported data on 48,457 participants from Latin America and the Caribbean region. Article distribution on the subject Periodontal Diseases on Quality of Life is presented in Figure 2, and the distribution by country is shown in Figure 3.

### Consensus of periodontal diseases and OHRQoL in LAC adult population

Untreated periodontal disease could cause a profound impact on individuals’ quality of life and revealed a complex interplay between oral health and general health. This interplay was supported by various studies across different countries in Latin America demonstrating the significance of addressing periodontal health as a crucial component of healthcare. In this review, 21 articles were identified for the relationship between periodontal diseases/conditions and quality of life in adult populations as depicted in Table 1.

**Table 1:** Publications on the associations between periodontal diseases and conditions on the Quality of life of Latin American and Caribbean adult population.

Author	Country	Sample size	Study design
Bandéca et al. (2011)^30^	Brazil	100	Cross-sectional
Collins et al. (2019)^24^	Dominican Republic, Jamaica, Puerto Rico	1821	Cross-sectional
Collins et al. (2024)^25^			Same population in both studies
De La Hoz Perafan et al., (2023)^15^	Colombia	229	Cross-sectional
Goergen et al. (2023)^17^	Brazil	1022	Cross-sectional
Goergen et al. (2021)^16^	Brazil	1465	Cohort
Rossi et al. (2023)^18^	Brazil	80	Cross-sectional
Llanos et al. (2018)^20^	Brazil	52	Cross-sectional
Lopes et al. (2009)^21^	Brazil	302	Cohort
Melo et al. (2016)^23^	Brazil	36	Cross-sectional
Mendez et al. (2017)^26^	Brazil	55	cohort
Meusel et al. (2015)^14^	Brazil	100	Cross-sectional
Mourão et al. (2015)^12^	Brazil	20	Case-control
Nascimento et al. (2021)^29^	Brazil	539	Cohort
Oliveira et al. (2020)^13^	Brazil	690	Cross-sectional
Palma et al. (2013)^11^	Brazil	150	Cross-sectional
Piedra-Hernández et al. (2023)^22^	Costa Rica	82	Cohort
Santuchi et al. (2016)^27^	Brazil	90	RCT
Wagner et al. (2016)^19^	Brazil	740	Cross-sectional
Passos-Soares et al. (2018)^31^	Brazil	306	Cross-sectional

In Brazil, studies by Palma et al.^11^ Mourão et al.,^12^ and Oliveira et al.^13^ demonstrated the detrimental effects of periodontal diseases on health-related quality of life, demonstrating how these conditions could lead to significant psychological and physical impairments. For example, Palma et al.,^11^ revealed that approximately 27% of the impact on health-related quality of life could be attributed to the severity of periodontal disease, self-perceived oral health, and the need for prosthetic rehabilitation. Furthermore, Meusel et al.^14^ in Brazil further highlighted the exacerbated effects of periodontitis in quality of life among individuals with low educational level. This underscored the importance of integrating oral health into broader health and social policies to improve health care and well-being. It is worth mentioning that Brazil is the most populated country at LAC having 223 million inhabitants with an excellent health care system in which basic oral health care is free for the whole people and in spite of those advantages the country is facing problems with coverage due to poverty and lack of education.

De La Hoz et al.^15^ in Colombia revealed that individuals with moderate periodontitis reported worse OHRQoL than those with mild or severe periodontitis forms, suggesting a complex relationship between disease severity and self-perceived quality of life. This finding underscored the need for comprehensive healthcare approaches encompassing periodontal health to improve overall well-being, particularly in Colombia, where the prevalence of periodontitis is high. Colombia is the second largest populated country in Latin America with 52 million inhabitants, in which specialized periodontal treatment is not available for most of the population.

Moreover, studies such as those by Goergen et al.^16,17^ in Brazil explored the broader relationship between oral conditions and quality of life, firstly associated with the staging grading based on the actual diagnostic criteria of periodontal diseases and secondly by encompassing a wide range of dental issues beyond traditional periodontal diseases among which xerostomia, halitosis, caries, and dentin hypersensitivity were significantly and directly associated with negative impacts on QOL. These studies contribute to a more comprehensive understanding of the impact of oral health on quality of life, by acknowledging the interconnectedness of various oral health conditions.

Further specificity was provided by studies examining the impact of periodontal diseases on particular segments of the population or specific conditions related to periodontal health. The research by Wagner et al.^18^ on gingival recession, Llanos et al.^19^ on aggressive and chronic periodontitis, Lopes et al.^20^ explored the specific link between chronic periodontitis and quality of life, and Piedra-Hernández et al.^21^ on dental anxiety post non-surgical treatment in Costa Rica. Melo et al.^22^ also focused on the impact of dentin sensitivity associated with chronic periodontitis on quality of life, main emphasized its manageability with appropriate treatment.

Two studies by Collins et al.^23,24^ explored the connection between gingival health, good oral hygiene habits such as toothbrushing, interdental hygiene practices that were associated with better oral health and self-perception OHRQoL among Caribbean adults in three main Caribbean cities. Factors such as smoking, infrequent dental visits, and chronic diseases were found to have significant impact on OHRQoL. Overall, this body of evidence offers nuanced views of varied dimensions of the impact of periodontal diseases on quality of life. These studies explore how different aspects of periodontal health, including gingival recession and dental anxiety, influence individuals’ daily experiences and overall well-being.

Specific research focusing on treatment methods and their efficacy further deepens this understanding. Mendez et al.^25^ and Santuchi et al.^27^ compared different periodontal treatment approaches, and revealed insights into how managing periodontal disease can improve quality of life. These findings emphasize the importance of effective treatment modalities in mitigating the adverse effects of periodontal diseases on OHRQOL.

Moreover, Ruano et al.^28^ investigated the impact of social factors and gingival bleeding effect on oral health-related quality of life. They discovered a significant correlation between lower individual social capital and poorer OHRQoL among adults using the Brazilian Unified Health System (SUS), pointing out the critical role of social determinants in oral health outcomes. This calls for policies that address social factors to enhance public health.

Finally, research such as that of Nascimento et al.^28^ showed the impact of clinical and self-reported oral conditions on quality of life, emphasizing the significant influence of self-perception in evaluating oral health outcomes. In this way, the research by Bandéca et al.^30^ in Brazil illustrated the relationship between self-perception of oral health and clinical factors, emphasizing the significant impact oral health has on individuals’ quality of life. This study contributed valuable insights into the dynamics between oral health, self-perception, and quality of life. This perspective is crucial for developing patient-centered care approaches that address clinical needs and individuals’ subjective experiences.

The predominance of cross-sectional studies among the articles reviewed highlights a limitation in establishing causality between periodontal diseases and impact in quality of life. This demonstrates the necessity for longitudinal research to understand the temporal dynamics of these relationships better. Additionally, there is a clear need for expanding research across diverse Latin American countries. These efforts will aid in developing integrated care strategies tailored to the varied cultures and populations in the region, ultimately improving health outcomes for individuals affected by periodontal diseases.

### Consensus of periodontal diseases and OHRQoL in LAC adolescents and schoolchildren

We identified 22 cross-sectional and cohort studies from Chile, Brazil, and Ecuador that reported the influence of periodontal diseases in adolescents, with a total population of 36,287 participants (Table 2).

**Table 2:** Publications on the associations between periodontal diseases and conditions on Quality of life of Latin American adolescents and preschool children population.

Author	Country	Sample size	Study summary and main findings
Balseca Ibarra et al. (2023)^34^	Ecuador	998	A cross-sectional study found that gingival bleeding negatively impacted OHRQoL, particularly emotional and social well-being.
Lattanzi et al. (2020)^47^	Brazil	319	A cross-sectional study found better OHRQoL in adolescents participating in a school health program.
Ortiz et al. (2020)^48^	Brazil	743	A cohort study found gingivitis at baseline was associated with higher overall and emotional OHRQoL scores at 2-year follow-up.
Sfreddo et al. (2019)^49^	Brazil	747	A 2-year cohort study found adolescents from lower SES backgrounds reported worse OHRQoL at follow-up.
Maia et al. (2018)^38^	Brazil	564	A cross-sectional study found dental caries and periodontal pockets negatively impacted OHRQoL, to a greater extent o in remote communities.
Maroneze et al. (2018)^50^	Brazil	67	A cross-sectional study found bleeding and gingival edema in the anterior region was associated with worse OHRQoL in adolescents.
Machry et al. (2018)^35^	Brazil	1,134	A cross-sectional study found that schoolchildren with gingival bleeding had higher OHRQoL scores.
Da Cunha et al. (2017)^42^	Brazil	5,402	A cross-sectional study found bleeding on probing and dental calculus was associated with impacted daily activities.
Kaminsky et al. (2016)^36^	Brazil	1,417	A cross-sectional study found a 49.6% prevalence of gingival bleeding in adolescents.
Alves et al. (2016)^33^	Brazil	119	Cross-sectional study found the presence of periodontal pockets and dental treatment needs were associated with worse OHRQoL in individuals with intellectual disabilities.
Vettore et al. (2016)^41^	Brazil	4,889	Cross-sectional study found gingivitis was one of the conditions resulting from clustering and predicted poor OHRQoL in children.
Vazquez et al. (2015)^51^	Brazil	1,172	Cross-sectional study found an increase in periodontal index associated with increased negative OHRQoL impact.
Schuch et al. (2015)^52^	Brazil	749	Cross-sectional study found dental plaque index >5 and severe malocclusion was associated with worse OHRQoL impact.
Tomoni et al. (2014)^53^	Brazil	1,134	Cross-sectional study found extensive-level gingivitis was associated with higher OHRQoL scores, even after adjusting for other factors.
Amato et al. (2014)^54^	Brazil	50	1-month longitudinal study found improvement in OHRQoL after an educational preventive program.
Peres et al. (2013)^40^	Brazil	5,445	Cohort study found gingival bleeding, dental calculus and periodontal pockets were associated with negative OHRQoL impact in adolescents.
Paula et al. (2012)^39^	Brazil	515	Cross-sectional study found bleeding was associated with worse OHRQoL in schoolchildren.
López & Belum (2007)^35^	Chile	9,203	Cross-sectional study found attachment loss and necrotizing ulcerative gingivitis were significantly associated with higher impact on OHRQoL in adolescents.
Núñez-Contreras et al. (2021)^43^	Chile	673	Cross-sectional study found no association between gingivitis and worsened quality of life in preschoolers of 3-5 years.
Barbosa et al. (2016)^45^	Brazil	167	A cross-sectional study found that gingivitis had no significant association with OHRQoL in children and preadolescents.
Barbosa et al. (2012)^44^	Brazil	145	A cross-sectional study found that bleeding had no impact on symptoms of anxiety or depression in pre-adolescents and school children.
Blazevic et al. (2008)^32^	Brazil	247	A cross-sectional study found no association between periodontal condition (CPI) and OHRQoL in the early adolescent population.

Studies have revealed a correlation between dental caries,^32^ gingival bleeding,^33-35^ and advanced forms of periodontal diseases that have substantial negative effects on the quality of life.^36-39^

A cross-sectional study of 9,203 Chilean high school students found that both attachment loss and necrotizing ulcerative gingivitis were significantly associated with higher impacts on oral health-related quality of life (OHRQoL) in adolescents.^37^

We identified three studies based on Brazilian national surveys. The first cohort study by Peres et al.^40^ analyzed data from 5,445 Brazilian adolescents aged 15–19 y/o who participated in the Brazilian National Oral Health Survey (SBBrasil, 2010) and found that gingival bleeding, dental calculus and periodontal pockets were associated with a negative impact on OHRQoL in adolescents.

The second study was a cross-sectional study by Vettore et al.,^41^ analyzing data on 7,208 children aged 12 years from the Brazilian Oral Health Survey (SBBrasil Project). The prevalence of dental caries and gingivitis was 41.5% and 26.6%, respectively. The prevalence of one or more Oral Impacts on Daily Performance (OIDP) items was 39.0% among children with gingivitis. The most prevalent performance influenced by all oral clinical conditions was ‘eating’.

The third was a cross-sectional study involving 5,402 adolescents from six macro-regions of São Paulo “SB São Paulo 2015” state survey,^40^ which reported multiple logistic regression for bleeding on probing (BoP) (OR = 1.45, 95%CI: 1.25–1.68; p < 0.01), the presence of dental calculus (OR = 1.55, 95%CI: 1.34–; p < 0.01). BoP and dental calculus are associated with impacted daily activities among adolescents.

Another cross-sectional study from Brazil with 247 adolescents reported that decayed teeth, despite the periodontal condition, correlated with worse OHRQoL.^30^

As regards the early childhood phase, a cross-sectional study with 673 Chilean children found no association between gingivitis and worsened quality of life in preschoolers aged 3–5 y/o.^43^ Similar results were reported on 559 pre-adolescent children from the Brazilian population.^32,44,45^

Children and adolescents with Down syndrome revealed a high prevalence of gingivitis at 91% and periodontitis in 33% of the individuals. Correlations between periodontal disease impact and clinical parameters such as bleeding on probing, probing depth, and attachment loss were statistically significant, affecting the quality of life. This highlights the considerable negative impact of periodontal disease on the daily lives of those with Down syndrome, exacerbating the severity of the condition.^46^

### Consensus of periodontal diseases, OHRQoL and diabetes in LAC

The intricate relationship between periodontal disease and diabetes mellitus (DM) is a subject of growing interest within research and clinical practice, given its profound implications for dental and systemic health and its significant impact on oral health-related quality of life (OHRQOL). Research, particularly in Latin America, has shown the bidirectional relationship between oral health and systemic conditions such as diabetes, demonstrating the broad influence of these interconnections on various life aspects.^55,56^ This body of evidence underscores the importance of holistic approaches to managing these conditions (Table 3)

**Table 3:** Publications on the influence of periodontal diseases and diabetes on quality of life in Latin American populations.

Author	Country	Sample size	Study design
De Pinho et al. (2012)^55^	Brazil	300	Cross-sectional
Drumond-Santana et al. (2007)^58^	Brazil	159	Cross-sectional
Morales et al. (2021)^59^	Chile	38	Cohort
Mourão et al. (2016)^56^	Brazil	500	Cross-sectional
Santos et al. (2020)^60^	Brazil	59	Cross-sectional
Sousa et al. (2019)^57^	Brazil	302	Cross-sectional

A study conducted by Pinho et al.^55^ in Brazil found that periodontal disease was significantly worse in prevalence, incidence, and severity among 300 diabetics, negatively impacting quality of life. Using OHIP-14 and various clinical criteria, they linked periodontal disease to functional limitation, pain, and psychological and physical disability, underscoring the need for integrated diabetes and periodontal health management. Further reinforcing this perspective, Mourão et al.^56^ in Brazil conducted a study where 250 chronic periodontitis (CP) patients with DM2 in comparison with 250 age and gender-matched controls without DM2 . The study found that DM2 significantly worsens the quality of life across all measured domains, including physical, social/family, functional, and emotional, even when diabetes was well-controlled. This points out the complex interaction between periodontal health and systemic diseases. Furthermore, the research suggested that even well-controlled DM2 combined with CP can negatively affect QoL. In line with these observations, Sousa et al.^57^ emphasized the critical role of oral health in the broader spectrum of diabetes management. Their study of 302 individuals in Brazil further established periodontitis as an important factor in diminishing the quality of life among those with type 2 diabetes, reinforcing the call for integrated healthcare interventions that encompass both diabetes and periodontal disease management.

Furthermore, Drumond-Santana et al.^58^ researched 159 diabetic individuals, highlighting the adverse impact of periodontal disease on quality of life and showing a significant correlation between periodontitis and negative quality of life outcomes. This study reinforced the necessity for specialized programs to mitigate the negative effects of periodontal disease on the quality of life of individuals with diabetes. This body of work collectively highlights the multifaceted challenges posed by the interplay between diabetes and periodontal health. More recently, Morales et al.,^59^ in Chile, during the COVID-19 pandemic, observed significant improvements in oral health and overall quality of life following periodontal therapy in 38 DM2 subjects. This finding suggests potential avenues for positive intervention, highlighting the transformative impact of periodontal care on the well-being of diabetic patients.

Whereas Santos et al.^60^ explored the relationship between chronic periodontitis and type 2 diabetes mellitus (DM2) in 59 subjects, with focus on polymorphisms in the vitamin D receptor gene. Moreover, participants’ quality of life (QoL) was assessed using the OHIP-14 questionnaire. Aspects such as psychological discomfort, physical pain, and physical disability were reported more frequently among individuals with DM2. However, the dimensions of the OHIP-14 in this study did not show positive associations between the severity of periodontitis and patients’ perception of quality of life.

The divergent findings across studies underscore the complexity of the relationship between periodontal disease, diabetes, and quality of life, demonstrating the necessity for further, diverse research in Latin America. Given the unique demographics and environmental factors of the region, well-designed border studies can provide crucial insights, informing effective, culturally tailored interventions. Expanding research across various Latin American countries is essential to develop integrated care strategies that improve health outcomes for affected individuals.

### Consensus of periodontal diseases, OHRQoL and pregnancy in LAC

Periodontal diseases have been hypothesized to increase the risk of Adverse Pregnancy Outcomes (APO) such as preterm birth and low birth weight. This association is biologically plausible due to the chronic inflammatory burden of periodontitis. Multiple meta-analyses have concluded that periodontitis increases the risk of preterm birth, low birth weight, and preeclampsia.^61-64^

Studies have consistently shown that the chronic inflammatory conditions associated with periodontal diseases can significantly impact not only oral health but also systemic health, influencing outcomes like preterm birth (PTB), low birth weight (LBW), and prolabor rupture of membranes (PROM). Highlighting this connection, research by Vogt et al.^65^ in Brazil among a cohort of 327 low-risk pregnant women found a clear association between periodontal disease and increased risks of adverse pregnancy outcomes. This underscores the vital need for integrating dental health within prenatal care programs to mitigate such risks, thus emphasizing the critical intersection between oral health and overall systemic well-being (Table 4).

**Table 4:** Publications on the influence of periodontal diseases and pregnancy on quality of life in Latin American populations.

Author	Country	Sample size	Study design
Caracho et al. (2020)^69^	Brazil	50	Cross-sectional
Cornejo et al. (2013)^68^	Argentina	80	Cross-sectional
Drumond-Santana et al. (2007)^58^	Brazil	159	Cross-sectional
Moimaz et al. (2016)^67^	Brazil	119	Cross-sectional
Musskopf et al. (2018)^64^	Brazil	210	RCT
Lopez ROSELL et al. (2013)^66^	Brazil	51	Cohort

Moreover, the significance of periodontal health extends beyond systemic implications to directly affect the quality of life of pregnant women. Studies by Lopez Rosell et al.^66^ and Moimaz et al.^67^ in Brazil have demonstrated that oral health conditions significantly affect the quality of life, particularly among pregnant women who exhibit a high prevalence of periodontal diseases. These findings call for a comprehensive approach to oral healthcare during pregnancy, and the need to address oral health not just for improving systemic health outcomes but also for its significant influence on the quality of life.

Moreover, research by Cornejo et al.^68^ in Buenos Aires, Argentina, investigated the oral health status of pregnant women from socially deprived populations. Their findings revealed a high prevalence of gingivitis (93.75%) and dental caries (92.1%). Nevertheless, interestingly, the perception of the impact of oral health on quality of life did not always align with the actual oral health status. This discrepancy indicates a complex relationship between oral health, systemic conditions, and quality of life perceptions, suggesting the need for increased awareness and education about oral health among pregnant women.

The association of periodontal diseases with systemic health conditions like obesity and hypertension during pregnancy has also been reported in Brazil by Caracho et al.^69^ Their study reported how systemic health challenges exacerbate periodontal conditions, adversely affecting pregnant women’s quality of life, and highlights the importance of integrating dental care into the broader prenatal healthcare framework to address these interconnected challenges effectively.

The positive impact of periodontal treatment on oral health-related quality of life (OHRQoL) among pregnant women was conclusively demonstrated in a randomized clinical trial by Musskopf et al.^64^ in Brazil. This trial showed significant improvements in OHRQoL for participants receiving periodontal treatment, underscoring the beneficial effects of oral health interventions on systemic health and overall quality of life.

Gestational Diabetes Mellitus (GDM) showed a 22.5% higher prevalence and severity of periodontitis. GDM was associated with a ten times higher risk of preterm birth, despite no difference in infants’ birth weight between groups in a cross-sectional study in Brazil.^70^ Relative to the plausibility of APO, a recent cross-sectional study examined the association between maternal overweight/obesity, periodontitis in late pregnancy, and infant birth weight in 100 Brazilian women.^71^ Maternal obesity may exacerbate pregnancy-related periodontal inflammation and contribute to poorer fetal growth outcomes. This sheds some light on the socioeconomic factors and the importance of controlling periodontal health to improve maternal and infant well-being.

These studies highlight a compelling case for integrating oral health care into prenatal care regimes across different contexts and populations. The evidence points towards a multifaceted relationship between periodontal diseases, systemic health conditions, and the quality of life of pregnant women, emphasizing the need for comprehensive healthcare strategies that include both oral and systemic health. Further research, especially in Latin American countries, is crucial to deepen our understanding of these relationships and to develop targeted interventions that can effectively improve the health outcomes for pregnant women and their babies.

### Consensus of periodontal diseases, OHRQoL and Smoking in LAC

Epidemiological studies showed a high prevalence of periodontal disease among smokers in Latin American and Caribbean populations.^72,73^ Epidemiological studies were mainly from Mexico, Brazil, Chile, Colombia, Uruguay, Argentina, and Peru, and they reported prevalence ranging from 35% to 90%.^74-76^ People with severe forms of periodontitis have been reported in 5% to 17% of adults, depending on the population.^66^ Smoking habits were identified as a significant risk factor associated with increased probability and severity of periodontitis.^73,74^

When considering gingivitis, three Latin American Cities, Mexico City (Mexico), Great Metropolitan Area (Costa Rica), and Bogota (Colombia), reported no correlation between smoking and plaque-induced gingivitis.^76^

When examining the impact of periodontal disease in conjunction with smoking on quality of life in Latin America, only one article was uncovered in the scoping review. Conducted in Brazil by Arruda et al.^77^ this study delved into the subject by focusing on the correlation between Oral Health-Related Quality of Life (OHRQoL) and periodontal status among individuals with varying smoking habits.

This cross-sectional study, conducted in Brazil, enrolled 100 participants and evaluated their periodontal health and OHRQoL using the OHIP-14 scale. The findings revealed that current smokers displayed significantly poorer periodontal health in terms of bleeding on probing (BOP), clinical attachment loss (CAL), and tooth count compared with never and former smokers. Despite these disparities in periodontal health, the overall impact on OHRQoL did not exhibit significant differences across smoking statuses. However, domains associated with social disability and handicap in the OHIP-14 were notably affected among current smokers.

This study underscores the intricate relationship between periodontal health, smoking, and quality of life, emphasizing the necessity for comprehensive oral health strategies that take into account patients’ smoking habits. It also underscores the need for further evidence to delve deeper into this condition and its implications for quality of life.

### Consensus of periodontal diseases, OHRQoL and Cardiovascular disease in LAC

The interaction of periodontal diseases and cardiovascular diseases has been explored elsewhere.^1^ Both diseases are known to be capable of significantly affecting an individual’s quality of life.^68^ With reference to research in Latin America, a study by Rebelo et al.^4^ in Brazil elucidated the complex relationship between periodontal status and systemic arterial hypertension, demonstrating how poor periodontal status mediated the relationship between socio-demographic factors, such as smoking and low income, and the oral health-related quality of life (OHRQoL) in hypertensive adults. This research showed the importance of comprehensive healthcare approaches that included the management of periodontal diseases to enhance the overall quality of life. Moreover, a study conducted by Taques et al.^79^ in Brazil also showed findings on the relationship between periodontal disease and quality of life in patients with circulatory diseases. Researchers found that elders and men showed greater periodontal disease severity, with the elderly also had a lower quality of life in functional capacity and physical aspects. However, the researchers could not find a correlation between the severity of periodontal disease and quality of life indicators.

The scarcity of research on the link between periodontal and cardiovascular diseases in Latin America and their impact on quality of life underscores a critical need for further regional studies. Addressing this gap is essential to improve public health strategies and enhance quality of life across the region.

## Discussion

The current consensus underscores the profound impact of periodontal and systemic diseases/conditions on Oral Health-Related Quality of Life (OHRQoL) among populations in Latin America and the Caribbean. The prevalent and severe nature of periodontal diseases, coupled with their significant association with systemic conditions such as diabetes, cardiovascular diseases, and adverse pregnancy outcomes, emphasizes the pressing need for comprehensive research and public health strategies in the region.

A recent study by Orlandi et al.^80^ investigated the effect of periodontitis treatment on various systemic health outcomes and pregnancy complications in randomized controlled trials with a minimum follow-up of 6 months. The meta-analysis revealed significant reductions in markers such as high-sensitivity C-reactive protein (his-CRP), interleukin (IL)-6, and plasma glucose, along with improvements in Flow-Mediated Dilation (FMD) and diastolic blood pressure after periodontitis treatment. Additionally, a protective effect on preterm deliveries (< 37 weeks) was observed. However, limited evidence was available regarding the impact on quality of life outcomes, emphasizing the need for further research to confirm the sustainability and universality of these benefits.

The relationship between periodontitis and comorbidities is multifaceted, involving common risk factors, pathophysiology, and bidirectional causal or non-causal relationships. Further well-designed randomized controlled clinical trials are needed to ascertain if periodontitis acts as a modifiable risk factor for associated comorbidities. Understanding these associations holistically may lead to novel therapeutic approaches to periodontitis treatment and management.

Integration of oral health into general health and social policies is crucial for effectively addressing the underlying causes and consequences of periodontal diseases. This review also advocates for the integration of periodontal care into the management of systemic diseases, by recognizing the bidirectional relationships between periodontal health and certain comorbidities.

Acknowledgement of the limitations of this scoping review is essential for understanding the scope and implications of its findings. While efforts were made to encompass studies from 33 Latin American and Caribbean countries, the distribution of research across these nations should be more balanced to better represent regional impacts. Additionally, the predominance of cross-sectional studies limits the ability to establish causality between periodontal disease, systemic conditions, and quality of life. Variations in demographics, healthcare systems, cultural values, and social determinants of health across the region may also affect the generalizability of the findings to certain populations within Latin America and the Caribbean.

## Conclusion

This scoping review provides a comprehensive overview of the burden of periodontal diseases in Latin America and the Caribbean and demonstrates that periodontal diseases is a critical public health issue in the region, with profound implications for societal and individual’s overall health and well-being. Quality of life is synergistically affected by the complex periodontitis-comorbidities that are concurrent and pathological interdependent and run along the life cycle and that recent research just started to understand and manage.

## Final considerations

The purpose of this Consensus was to provide a comprehensive overview of the latest research on the impact of periodontal diseases on Oral Health-Related Quality of Life (OHRQoL) and systemic diseases and conditions among people in countries from Latin America and the Caribbean (LAC). The region, characterized by its cultural, ethnic, and linguistic diversity, grapples with some of the highest levels of inequities and inequalities in health and education globally, posing challenges in description, understanding, and approach. Furthermore, poor management of political, economic, and public health services constrains the ability of governments to provide the entire population with equitable and quality health coverage, particularly for periodontal procedures.^28^

OHRQoL, defined as a multidimensional construct encompassing subjective evaluations of oral health, functional and emotional well-being, and satisfaction in daily life relative to oral health, presents complexities in interpretation and extrapolation across population groups from different countries.^81^ In a recent systematic review conducted in LAC, the impact of oral diseases on OHRQoL was explored, revealing that the majority of studies identified a significant impact on OHRQoL in children, adolescents, and adults with oral diseases, including periodontal diseases. Moreover, greater severity of oral disease correlated with a more pronounced impact on OHRQoL.^82^

Changing individual behaviors for improved oral health and implementing oral health education are considered pivotal yet challenging elements for enhancing knowledge levels and promoting health. These efforts may reduce the risk of periodontal diseases and their complications, thus fostering long-term periodontal and systemic health and overall well-being.^83,84^

Recent research in the region investigated the relationship between periodontal health knowledge and OHRQoL among Caribbean adults. The findings indicated that individuals with limited knowledge about gum health were more likely to report poorer OHRQoL compared with those who had a higher level of knowledge, highlighting the importance of promoting appropriate attitudes, practices, knowledge, and self-awareness of oral health to enhance both the oral health and quality of life of individuals in the region.^24^

### Future recommendations for research in the LAC region:

Increase representation of epidemiological studies across different countries in Latin America and the Caribbean (LAC) that explore the relationship between periodontal diseases and Oral Health-Related Quality of Life (OHRQoL). This would provide a more comprehensive understanding of how these conditions impact individuals in diverse cultural and socioeconomic contexts.

Conduct multicenter longitudinal studies in the region to investigate the associations between periodontitis and non-communicable diseases (NCDs), such as cardiovascular diseases, diabetes, and respiratory diseases. Longitudinal research can offer insights into the long-term effects and potential causal relationships between periodontal health and systemic conditions.

Focus research efforts on examining the impact of periodontal diseases on the quality of life of vulnerable populations, including those facing geographical limitations and limited access to oral health services. Understanding how these populations are affected can provide information on how best to target interventions and policies to address disparities in oral health outcomes.

Conduct intervention studies to assess the cost-effectiveness and impact of periodontal treatment on systemic conditions, particularly cardiovascular diseases, adverse pregnancy outcomes, diabetes, and respiratory diseases. Evaluating the benefits of periodontal care in managing these systemic conditions can provide information to guide healthcare decision-making and resource allocation.

Undertake a systematic evaluation of inequalities and inequities in oral health across Latin America and the Caribbean. By identifying disparities in access to oral health services, treatment outcomes, and oral health outcomes, policymakers can implement targeted actions, policies, and programs to reduce inequities and improve oral health equity in the region.

Promote interdisciplinary initiatives involving both public and private sectors to implement programs with the aim of increasing individual knowledge and self-awareness in oral health in the region. Collaborative efforts can leverage resources and expertise from multiple sectors to develop comprehensive oral health promotion strategies that address the diverse needs of populations across Latin America and the Caribbean.
